# Supplementary material for: Collective relational inference for learning heterogeneous interactions
Source: Nat Commun. 2024 Apr 12;15:3191. doi: 10.1038/s41467-024-47098-7 (PMC11258243; doi:10.1038/s41467-024-47098-7)
Supplement: Supplementary file 1 — Supplementary Information [file 41467_2024_47098_MOESM1_ESM.pdf]

Supplementary Information: Collective Relational Inference for learning heterogeneous interactions

Zhichao Han[1] **Olga Fink**[2†] **David S. Kammer**[1†*]

[1]Institute for Building Materials, ETH Zürich, 8093 Zürich, Switzerland
[2]Laboratory of Intelligent Maintenance and Operations Systems, EPFL, 1015 Lausanne, Switzerland

[*]To whom correspondence should be addressed. Email: `dkammer@ethz.ch`
[†]Joint supervision

1 Supplementary Notes

The important notations used in the paper are summarized in Supplementary Table 1.

Supplementary Table 1: Important symbols

**notation**	**meaning**
$G = (V, E)$	graph representation of the interacting system
$V = \{v_1, v_2, \ldots, v_{\|V\|}\}$	the set of nodes corresponding to entities in the interacting system, where v_i is i-th entity
$E = \{e_{i,j} : v_i, v_j \in V, i \neq j\}$	the set of edges corresponding to pairwise interactions, where $e_{i,j}$ is the directed edge from v_j to v_i
$S_{(i)}$	the subgraph associated with v_i
$\Gamma^t(i)$	the neighbors of v_i at time t
$\Gamma(i) = \bigcup_t \Gamma^t(i)$	the neighbors of v_i across all time steps
K	the number of different interactions in a heterogeneous system
d	the spatial dimension
$\mathbf{r}_i^t \in \mathbb{R}^d$	(for particle systems) position of v_i at time t
$\dot{\mathbf{r}}_i^t \in \mathbb{R}^d$	(for particle systems) velocity of v_i at time t
$\ddot{\mathbf{x}}_i^t \in \mathbb{R}^d$	ground-truth state increment of v_i at time t (*e.g.*, the acceleration), which is used to update the state $\mathbf{x}_i^{t+1}$ from $\mathbf{x}_i^t$
$\hat{\ddot{\mathbf{x}}}_i^t \in \mathbb{R}^d$	predicted state increment of particle v_i at time t
$m_i \in \mathbb{R}$	(for particle systems) mass of particle v_i, it is a constant
$\mathbf{x}_i^t$	feature vector of node v_i at time t
$\mathrm{z}_{i,j}$	the categorical random variable of the edge $e_{i,j}$
$\mathrm{z}_{(i)}$	the categorical random variable of the subgraph $S_{(i)}$ in CRI
$\phi_{\mathrm{z}_{(i)}}(j) \in \{1, 2, \ldots, K\}$	the $\mathrm{z}_{i,j}$ of $e_{i,j}$ given the realization $z_{(i)}$ of $S_{(i)}$ in CRI
z	the value of a random variable
$\Theta = \{\theta_1, \theta_2, \ldots, \theta_K\}$	learnable parameters of K neural networks
$\mathcal{N}(\mathbf{x} \mid \boldsymbol{\mu}, \boldsymbol{\Sigma})$	Gaussian distribution over $\mathbf{x}$ with mean $\boldsymbol{\mu}$ and covariance $\boldsymbol{\Sigma}$
π_z	the prior probability of a subgraph having realization z
$\boldsymbol{\pi} = \{\pi_1, \pi_2, \ldots, \pi_M\}$	the prior probabilities of a subgraph with different realizations
τ_k	the prior probability that $e_{i,j}$ having realization $\mathrm{z}_{i,j} = z$
$\boldsymbol{\tau} = \{\tau_1, \tau_2, \ldots, \pi_K\}$	the prior probabilities of edge types
Υ	the set of all possible realizations
σ^2	the pre-defined variance for the multivariate normal distributions
ω_z	the prior probability that any group in Var-CRI having realization z
$\boldsymbol{\omega} = \{\omega_1, \ldots, \omega_{\|g\|}\}$	the prior probabilities of all realizations for a group in Var-CRI ($\|g\|$ is the maximal group size)

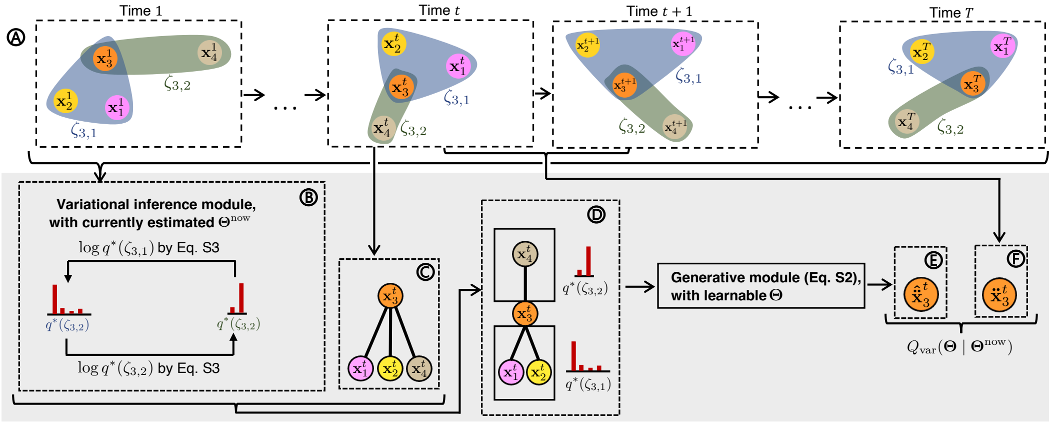

Supplementary Figure 1: **Framework of Var-CRI.** (A) The particle system over time. At each time step, every node is described by the feature vector $\mathbf{x}_i^t$, representing its state. (B) The variational inference module replaces the exact inference module in CRI. We divide the incoming edges of v_3 into two disjoint groups and infer the probabilities $q^*(\zeta_{3,1})$ and $q^*(\zeta_{3,2})$. (C) The subgraph $S_{(3)}$ at time t. (D) The subgraph $S_{(3)}$ with different realizations of its two groups at time t, which are the input of the generative module. (E) The predicted state increment of v_3 at time t which is the expectation over $q^*(\zeta_{3,1})$ and $q^*(\zeta_{3,2})$. (F) The ground-truth state increment computed by looking at particle states at two consecutive time steps.

2 Supplementary Methods

2.1 Variational Collective Relational Inference (Var-CRI)

The computation complexity of CRI limits its applicability in cases where each particle has too many interactions. Such high complexity arises from the exact computation of the expectation in Eq. 5. In fact, computing the expectation of high dimensional discrete variables is a general problem and the main effort in Bayesian inference is developing techniques to approximate such expectation [1]. These approximation techniques can be broadly divided into two categories: Monte Carlo method [2] and the variational method [3]. Here, we use the variational method as one example to approximate the expectation. Please note that finding a better way to approximate the expectation is not the focus of this paper. Many advanced methods [4–6] can be considered for achieving a better approximation of the expectation.

The underlying idea is that we partition the incoming edges of each particle into several disjoint groups and we assume the edge types of edges in different groups are independent. Suppose we partition the incoming edges of v_i into M groups, we use random variables $\zeta_{i,1}$, ..., $\zeta_{i,M}$ to denote the edge types in these M groups. For example, in Supplementary Fig. 1, supposing particle v_3 has three incoming edges $e_{3,1}$, $e_{3,2}$ and $e_{3,4}$. The random variables associated with these three edges are $\mathrm{z}_{3,1}$, $\mathrm{z}_{3,2}$ and $\mathrm{z}_{3,4}$. We partition these three incoming edges of v_3 into two groups and these two groups can be $\zeta_{3,1} = \{\mathrm{z}_{3,1}, \mathrm{z}_{3,2}\}$ and $\zeta_{3,2} = \{\mathrm{z}_{3,4}\}$. Again, we use $\phi_{\zeta_{i,1},\ldots,\zeta_{i,M}}(i,j) \in \{1, 2, \ldots, K\}$ to map the block realizations $\zeta_{i,1}$, ..., $\zeta_{i,M}$ to the interaction type $\mathrm{z}_{i,j}$ of edges $e_{i,j}$ ($\forall j \in \Gamma(i)$). The predicted state increment of v_i ($\forall i$) given $\zeta_{i,1}, \ldots, \zeta_{i,M}$ is:

$$\text{Using standard message-passing GNN:} \quad \hat{\dot{\mathbf{x}}}^t_{i|\zeta_{i,1},\ldots,\zeta_{i,M}} = \mathrm{NN}_{\text{node}}\left(\sum_{j\in\Gamma(i)} \mathrm{NN}^{\phi_{\zeta_{i,1},\ldots,\zeta_{i,M}}(i,j)}(\mathbf{x}_i^t, \mathbf{x}_j^t)\,,\ \mathbf{x}_i^t\right)$$
$$\text{Or using PIG'N'PI for particles:} \quad \hat{\dot{\mathbf{x}}}^t_{i|\zeta_{i,1},\ldots,\zeta_{i,M}} = \sum_{j\in\Gamma(i)} \mathrm{NN}^{\phi_{\zeta_{i,1},\ldots,\zeta_{i,M}}(i,j)}(\mathbf{x}_i^t, \mathbf{x}_j^t)/m_i \tag{1}$$

where $\mathrm{NN}_{\text{node}}$ is a neural network that takes the incoming interactions and the state of itself as input.

The conditional likelihood given the realizations of blocks is computed by fitting the ground-truth state increment into the multivariate normal distribution whose center is the predicted state increment by Eq. 1 given the block realizations $\zeta_{i,1}, \ldots, \zeta_{i,M}$:

$$l(\Theta \mid \ddot{\mathbf{x}}_i^t, \zeta_{i,1}, \ldots, \zeta_{i,M}) = p(\ddot{\mathbf{x}}_i^t \mid \Theta, \zeta_{i,1}, \ldots, \zeta_{i,M}) = \mathcal{N}\left(\ddot{\mathbf{x}}_i^t \mid \hat{\ddot{\mathbf{x}}}_{i|\zeta_{i,1},\ldots,\zeta_{i,M}}^t, \sigma^2 \boldsymbol{I}\right) \tag{2}$$

We denote by ω_z the prior probability that any group $\zeta_{i,j}$ having realization z and $\boldsymbol{\omega} = \{\omega_1, \ldots, \omega_{|g|}\}$ ($|g|$ is the maximal group size). The learnable variables in Var-CRI are $\boldsymbol{\Theta} = (\Theta, \boldsymbol{\omega})$.

In the expectation (E) step of Var-CRI, we seek distributions $q^*(\zeta_{i,j})$ ($j = 1, 2, \ldots, M$) such that $q^*(\zeta_i) = \prod_j q^*(\zeta_{i,j})$ approximates the current estimation of the posterior $p(\mathrm{z}_{(i)} = z \mid \ddot{\mathbf{x}}_i^{1:T}, \boldsymbol{\Theta}^{\text{now}})$ in Eq. 4. One can show [3]:

$$\log q^*(\zeta_{i,j}) \propto \mathbb{E}_{-j}\left[\log p(\zeta_{i,j}, \zeta_{i,-j} \mid \ddot{\mathbf{x}}_i^{1:T}, \boldsymbol{\Theta}^{\text{now}})\right] \tag{3}$$

where the expectation $\mathbb{E}_{-j}$ integrates over the remaining factor $q^*(\zeta_{i,j'})$, $\forall j' \neq j$. As q^* has no closed solution, we adopt the same method as in [3] to compute q^* by initializing $q^*(\zeta_{i,j})$ uniformly and cycling through expectation to update each factor. The posterior $p(\zeta_{i,1}, \ldots, \zeta_{i,M} \mid \ddot{\mathbf{x}}_i^{1:T}, \boldsymbol{\Theta}^{\text{now}})$ in Eq. 3 is computed by Bayes rule as shown in Eq. 4:

$$\begin{aligned} &p(\zeta_{i,1} = z_1, \ldots, \zeta_{i,M} = z_M \mid \ddot{\mathbf{x}}_i^{1:T}, \boldsymbol{\Theta}^{\text{now}}) \\ =&\frac{\omega_{z_1} \ldots \omega_{z_M} \prod_{t=1}^{T} p(\ddot{\mathbf{x}}_i^t \mid \zeta_{i,1} = z_1, \ldots, \zeta_{i,M} = z_M, \Theta^{\text{now}})}{\sum_{z'_1,\ldots,z'_M} \omega_{z_1} \ldots \omega_{z_M} \prod_{t=1}^{T} p(\ddot{\mathbf{x}}_i^t \mid \zeta_{i,1} = z'_1, \ldots, \zeta_{i,M} = z'_M, \Theta^{\text{now}})} \end{aligned} \tag{4}$$

where $p(\ddot{\mathbf{x}}_i^t \mid \zeta_{i,1} = z_1, \ldots, \zeta_{i,M} = z_M, \Theta^{\text{now}})$ is computed by Eq. 1 and Eq. 2.

The Q function for Var-CRI becomes

$$\begin{aligned} Q_{\text{var}}(\boldsymbol{\Theta} \mid \boldsymbol{\Theta}^{\text{now}}) = &\sum_{i=1}^{|V|} \sum_{j=1}^{M} \mathbb{E}_{\zeta_{i,j} \sim q^*(\zeta_{i,j})}\left[\log \omega_{\zeta_{i,j}}\right] \\ &+ \sum_{i=1}^{|V|} \mathbb{E}_{\zeta_{i,1},\ldots,\zeta_{i,M} \sim q^*(\zeta_{i,1}) \ldots q^*(\zeta_{i,M})}\left[\sum_{t=1}^{T} \log p(\ddot{\mathbf{x}}_i^t \mid \Theta, \zeta_{i,1}, \ldots, \zeta_{i,M})\right] \end{aligned} \tag{5}$$

In the maximization (M) step, we update our estimation to the prior $\boldsymbol{\omega}$ and the neural network parameters Θ by maximizing the Q_{var} in Eq. 5. Similar to the CRI, $\boldsymbol{\omega}$ has the analytic solution but Θ does not. We again take one gradient ascent step to update Θ. In addition, since the posterior of ground-truth state increment $p(\ddot{\mathbf{x}}_i^t \mid \Theta, \zeta_{i,1}, \ldots, \zeta_{i,M})$ is a multivariate normal distribution whose center is the predicted state increment, the $\log p(\ddot{\mathbf{x}}_i^t \mid \Theta, \zeta_{i,1}, \ldots, \zeta_{i,M})$ has a quadratic form of the predicted pairwise forces which are approximated by these K neural networks. The quadratic form and the independence between different groups result in the complexity $\mathcal{O}(MK^{\lceil|\Gamma(i)|/M\rceil})$ in computing the expectation over M blocks $\mathbb{E}_{\zeta_{i,1},\ldots,\zeta_{i,M} \sim q^*(\zeta_{i,1}) \ldots q^*(\zeta_{i,M})}\left[\sum_t \log p(\ddot{\mathbf{x}}_i^t \mid \zeta_1, \ldots, \zeta_M; \Theta)\right]$.

2.2 Detailed derivation and training process of CRI

Here, we provide the detailed derivation and training strategy of the proposed CRI method (Sec. 4.2). The derivations of Var-CRI (SI Sec. 2.1) and Evolving-CRI (Sec. 4.3) follow the same approach.

We use the same notations as in Sec. 4.2. Our goal is to optimize the learnable parameters by maximizing the marginal likelihood given the ground-truth state increment, as defined in Eq. 3 in Sec. 4.2

$$\begin{aligned} L(\mathbf{\Theta}) &= \prod_{i=1}^{|V|} \sum_{\mathrm{z}_{(i)}} p(\ddot{\mathbf{x}}_i^{1:T}, \mathrm{z}_{(i)} \mid \Theta) = \prod_{i=1}^{|V|} \sum_{z=1}^{|\Upsilon|} \underbrace{p(\mathrm{z}_{(i)} = z)}_{\pi_z} p(\ddot{\mathbf{x}}_i^{1:T} \mid \Theta, \mathrm{z}_{(i)} = z) \\ &= \prod_{i=1}^{|V|} \sum_{z=1}^{|\Upsilon|} \underbrace{p(\mathrm{z}_{(i)} = z)}_{\pi_z} \prod_{t=1}^{T} l(\Theta \mid \ddot{\mathbf{x}}_i^t, \mathrm{z}_{(i)} = z) \end{aligned}$$

By explicitly expressing $l(\Theta \mid \ddot{\mathbf{x}}_i^t, \mathrm{z}_{(i)})$, as defined by Eq. 2, the marginal likelihood can be written as:

$$L(\mathbf{\Theta}) = \prod_{i=1}^{|V|} \sum_{z=1}^{|\Upsilon|} \pi_z \left[\prod_{t=1}^{T} \mathcal{N}\left(\ddot{\mathbf{x}}_i^t \mid \hat{\ddot{\mathbf{x}}}_{i|\mathrm{z}_{(i)}}^t, \sigma^2 \boldsymbol{I} \right) \right] \tag{6}$$

Directly optimizing $\log L(\mathbf{\Theta})$ with respect to $\mathbf{\Theta} = (\Theta, \boldsymbol{\pi})$ is intractable because of the summation in the logarithm. Therefore, we optimize the model under the generalized EM procedure [7].

In the expectation step, we compute the complete-data likelihood function, and the posterior distribution of latent variables given the ground-truth state increment and current estimations of the learnable parameters. The complete-data likelihood function $L(\mathbf{\Theta}; \ddot{\mathbf{x}}, Z)$, where $\ddot{\mathbf{x}} = \{\ddot{\mathbf{x}}_1^{1:T}, \dots, \ddot{\mathbf{x}}_{|V|}^{1:T}\}$ is the ground-truth state increment of all particles across different time steps and $Z = \{\mathrm{z}_{(1)}, \dots, \mathrm{z}_{(|V|)}\}$ contains the latent variables of all subgraphs, is:

$$\begin{aligned} L(\mathbf{\Theta} \mid \ddot{\mathbf{x}}, Z) &= p(\ddot{\mathbf{x}}, Z \mid \mathbf{\Theta}) \\ &= \prod_{i=1}^{|V|} \prod_{z=1}^{|\Upsilon|} \left[\pi_z p(\ddot{\mathbf{x}}_i^{1:T} \mid \Theta, \mathrm{z}_{(i)} = z) \right]^{\mathbb{I}(\mathrm{z}_{(i)} = z)} \\ &= \prod_{i=1}^{|V|} \prod_{z=1}^{|\Upsilon|} \left[\pi_z \prod_{t=1}^{T} \mathcal{N}\left(\ddot{\mathbf{x}}_i^t \mid \hat{\ddot{\mathbf{x}}}_{i|\mathrm{z}_{(i)}}^t, \sigma^2 \boldsymbol{I} \right) \right]^{\mathbb{I}(\mathrm{z}_{(i)} = z)} \end{aligned} \tag{7}$$

where $\mathbb{I}(x)$ is the indicator function such that $\mathbb{I}(x)$ is equal to 1 if x is true, and 0 if x is false.

Then, the log complete-data likelihood is given by:

$$\begin{aligned} \log L(\mathbf{\Theta} \mid \ddot{\mathbf{x}}, Z) &= \sum_{i=1}^{|V|} \sum_{z=1}^{|\Upsilon|} \log \left[\pi_z \prod_{t=1}^{T} \mathcal{N}\left(\ddot{\mathbf{x}}_i^t \mid \hat{\ddot{\mathbf{x}}}_{i|\mathrm{z}_{(i)}=z}^t, \sigma^2 \boldsymbol{I} \right) \right]^{\mathbb{I}(\mathrm{z}_{(i)} = z)} \\ = \sum_{i=1}^{|V|} \sum_{z=1}^{|\Upsilon|} \mathbb{I}(\mathrm{z}_{(i)} = z) & \left[\log \pi_z + \sum_{t=1}^{T} \left(- \frac{\sum_{j=1}^{d} (\ddot{\mathbf{x}}_i^t[j] - \hat{\ddot{\mathbf{x}}}_{i|\mathrm{z}_{(i)}=z}^t[j])^2}{2\sigma^2} - \frac{d}{2} \log(2\pi) - d \log \sigma \right) \right] \end{aligned} \tag{8}$$

where the predicted state increment $\hat{\ddot{\mathbf{x}}}_{i|\mathrm{z}_{(i)}=z}^t$ is computed by Eq. 1. $\ddot{\mathbf{x}}_i^t[j]$ and $\hat{\ddot{\mathbf{x}}}_{i|\mathrm{z}_{(i)}=z}^t[j]$ are the j-th element in the vector $\ddot{\mathbf{x}}_i^t$ and $\hat{\ddot{\mathbf{x}}}_{i|\mathrm{z}_{(i)}=z}^t$, respectively, and d is the spatial dimension.

The mixing coefficient π_z ($z = 1, 2, \dots, |\Upsilon|$), which is the *prior* probability of the realizations of the subgraph, can be expressed as the product of the *prior* probability of the interaction type of each edge in this subgraph, given that the interaction types of the edges are independent variables *a priori*. Using the same notation as in Sec. 4.3, we denote by τ_k the *prior* probability that one edge has the k-th interaction type ($k \in \{1, 2, \dots, K\}$) and the prior distribution is $\boldsymbol{\tau} = (\tau_1, \tau_2, \dots, \tau_K)$. Let $C_z(k)$ be the number of edges in a subgraph having k-th interaction type, given that this

Algorithm 1: CRI Training

Randomly initialize $\mathbf{\Theta}$ as $\mathbf{\Theta}^{\text{now}} = (\boldsymbol{\tau}^{\text{now}}, \Theta^{\text{now}})$;
repeat
  # E-step
  Compute $Q_{\text{CRI}}(\mathbf{\Theta} \mid \mathbf{\Theta}^{\text{now}})$ by Eq. 10 where π_z is computed by Eq. 9, $\hat{\ddot{\mathbf{x}}}$ is computed by Eq. 1, $p(\mathrm{z}_{(i)} = z \mid \ddot{\mathbf{x}}_i^{1:T}, \mathbf{\Theta}^{\text{now}})$ is computed by Eq. 4;
  # M-step
  $\{\tau_1^{new}, \dots, \tau_K^{new}\} \leftarrow \arg\max_{\tau_1,\dots,\tau_K} Q_{\text{CRI}}(\mathbf{\Theta} \mid \mathbf{\Theta}^{\text{now}})$ by Eq. 12;
  $\theta_k^{new} \leftarrow \theta_k^{\text{now}} + \text{step\_size} \cdot \partial Q_{\text{CRI}} / \partial \theta_k$ $(\forall k)$ by Eq. 13;
  # Update the current estimation of $\mathbf{\Theta}$.
  $\boldsymbol{\tau}^{\text{now}} \leftarrow \boldsymbol{\tau}^{new}$;
  $\Theta^{\text{now}} \leftarrow \Theta^{new}$;
until *converge or reach max training epochs*;

subgraph has z-th realization. For example, for the first realization in Fig. 7B, $C_{\text{“r1”}}(\text{“blue”}) = 2$ and $C_{\text{“r1”}}(\text{“green”}) = 0$. Then, the relation between $\boldsymbol{\pi}$ and $\boldsymbol{\tau}$ is expressed by:

$$\forall z: \quad \pi_z = \tau_1^{C_z(1)} \tau_2^{C_z(2)} \cdots \tau_K^{C_z(K)} \tag{9}$$

With the posterior probability $p(\mathrm{z}_{(i)} \mid \ddot{\mathbf{x}}_i^{1:T}, \mathbf{\Theta}^{\text{now}})$, which is indicated by Eq. 4, and the log complete-data likelihood $\log L(\mathbf{\Theta} \mid \ddot{\mathbf{x}}, Z)$ from Eq. 8, we can write the Q function of CRI with $Q_{\text{CRI}}(\mathbf{\Theta} \mid \mathbf{\Theta}^{\text{now}}) = \sum_{i=1}^{|V|} \mathbb{E}_{\mathrm{z}_{(i)} \sim p(\mathrm{z}_{(i)} \mid \ddot{\mathbf{x}}_i^{1:T}, \mathbf{\Theta}^{\text{now}})} \left[p(\ddot{\mathbf{x}}_i^{1:T}, \mathrm{z}_{(i)} \mid \mathbf{\Theta}) \right]$ as already given by Eq. 5 but repeated here for completeness:

$$\begin{aligned} Q_{\text{CRI}}(\mathbf{\Theta} \mid \mathbf{\Theta}^{\text{now}}) = & \sum_{i=1}^{|V|} \sum_{z=1}^{|\Upsilon|} p(\mathrm{z}_{(i)} = z \mid \ddot{\mathbf{x}}_i^{1:T}, \mathbf{\Theta}^{\text{now}}) \log \pi_z \\ & + \sum_{i=1}^{|V|} \sum_{z=1}^{|\Upsilon|} p(\mathrm{z}_{(i)} = z \mid \ddot{\mathbf{x}}_i^{1:T}, \mathbf{\Theta}^{\text{now}}) \sum_{t=1}^{T} \left(- \frac{\sum_{j=1}^{d} (\ddot{\mathbf{x}}_i^t[j] - \hat{\ddot{\mathbf{x}}}_{i|\mathrm{z}_{(i)}=z}^t[j])^2}{2\sigma^2} \right) \\ & + \sum_{i=1}^{|V|} \sum_{z=1}^{|\Upsilon|} p(\mathrm{z}_{(i)} = z \mid \ddot{\mathbf{x}}_i^{1:T}, \mathbf{\Theta}^{\text{now}}) \sum_{t=1}^{T} \left[-\frac{d}{2} \log(2\pi) - d \log \sigma \right] \end{aligned} \tag{10}$$

In the maximization step, we update the learnable parameters $\mathbf{\Theta} = (\boldsymbol{\pi}, \Theta)$ from currently estimated values $\mathbf{\Theta}^{\text{now}} = (\boldsymbol{\pi}^{\text{now}}, \Theta^{\text{now}})$ to $\Theta^{new} = (\boldsymbol{\pi}^{new}, \Theta^{new})$ by optimizing the $Q_{\text{CRI}}(\mathbf{\Theta} \mid \mathbf{\Theta}^{\text{now}})$ in Eq. 10. The first term on right-hand side of Eq. 10 only depends on the mixing coefficients $\{\pi_1, \dots, \pi_{|\Upsilon|}\}$, which are functions of $\{\tau_1, \dots, \tau_K\}$ (see Eq. 9). The second term only depends on $\{\theta_1, \dots, \theta_K\}$ since the predicted state increment is the function of $\{\theta_1, \dots, \theta_K\}$ (see Eq. 1). The third term is a constant with respect to the pre-defined σ^2. Therefore, we can optimize $\{\tau_1, \dots, \tau_K\}$ and $\{\theta_1, \dots, \theta_K\}$ separately.

We can further write the first term in Eq. 10 as

$$\begin{aligned}
&\sum_{i=1}^{|V|}\sum_{z=1}^{|\Upsilon|} p(\mathrm{z}_{(i)} = z \mid \ddot{\mathbf{x}}_i^{1:T}, \mathbf{\Theta}^{\mathrm{now}}) \log \pi_z \\
=&\sum_{i=1}^{|V|}\sum_{z=1}^{|\Upsilon|} p(\mathrm{z}_{(i)} = z \mid \ddot{\mathbf{x}}_i^{1:T}, \mathbf{\Theta}^{\mathrm{now}}) \left(C_z(1)\log\tau_1 + \cdots + C_z(K)\log\tau_K\right) \\
=&\sum_{i=1}^{|V|}\left[\sum_{z=1}^{|\Upsilon|} p(\mathrm{z}_{(i)} = z \mid \ddot{\mathbf{x}}_i^{1:T}, \mathbf{\Theta}^{\mathrm{now}}) C_z(1)\right]\log\tau_1 + \cdots + \sum_{i=1}^{|V|}\left[\sum_{z=1}^{|\Upsilon|} p(\mathrm{z}_{(i)} = z \mid \ddot{\mathbf{x}}_i^{1:T}, \mathbf{\Theta}^{\mathrm{now}}) C_z(K)\right]\log\tau_K \\
=&\sum_{i=1}^{|V|}\chi_{i1}^{\mathrm{now}}\log\tau_1 + \cdots + \sum_{i=1}^{|V|}\chi_{iK}^{\mathrm{now}}\log\tau_K
\end{aligned} \tag{11}$$

where $\chi_{ik}^{\mathrm{now}} = \sum_{z=1}^{|\Upsilon|} p(\mathrm{z}_{(i)} = z \mid \ddot{\mathbf{x}}_i^{1:T}, \mathbf{\Theta}^{\mathrm{now}}) C_z(k)$ is the expected number of edges whose interaction type is k in $S_{(i)}$, given $\mathrm{z}_{(i)} = z$, with respect to the current estimation of the posterior probability $p(\mathrm{z}_{(i)} \mid \ddot{\mathbf{x}}_i^{1:T}, \mathbf{\Theta}^{\mathrm{now}})$. Eq. 11 has the same form as the MLE for the multinomial distribution. Therefore, $\arg\max_{\tau_1,\ldots,\tau_k} Q_{\mathrm{CRI}}(\mathbf{\Theta} \mid \mathbf{\Theta}^{\mathrm{now}})$ will be

$$\tau_k^{new} \leftarrow \frac{\sum_{i=1}^{|V|}\chi_{ik}^{\mathrm{now}}}{\sum_{i=1}^{|V|}\sum_{k'=1}^{K}\chi_{ik'}^{\mathrm{now}}} \tag{12}$$

For the parameters $\{\theta_1 \ldots, \theta_K\}$, we cannot compute $\arg\max_{\theta_1,\ldots,\theta_K} Q_{\mathrm{CRI}}(\mathbf{\Theta} \mid \mathbf{\Theta}^{\mathrm{now}})$ analytically, hence we take one gradient ascent step to update $\{\theta_1, \ldots, \theta_K\}$:

$$\theta_k^{new} \leftarrow \theta_k^{\mathrm{now}} + \text{step\_size} \cdot \partial Q_{\mathrm{CRI}} / \partial\theta_k \tag{13}$$

We iteratively apply the expectation-step and maximization-step to optimize the parameters until the model converges to the (local) minimum or reaches the maximal training epochs. We denote by $\mathbf{\Theta}^*$ the parameters after training. The pseudocode of training procedures is summarized in Algorithm 1. After training, we infer the interaction type for each subgraph $S_{(i)}$ by $\mathrm{z}_{(i)}^* = \arg\max_z p(\mathrm{z}_{(i)} = z \mid \ddot{\mathbf{x}}_i^{1:T}, \mathbf{\Theta}^*)$. The inferred interaction type for every edge $e_{i,j}$ is obtained by the mapping $\phi_{\mathrm{z}_{(i)}^*}(j)$ as defined in Sec. 4.2.

3 Supplementary Discussion

3.1 Hyperparameters of CRI, Var-CRI and Evolving-CRI for different experiments

We empirically find the depth of NN^1, ..., NN^K and the Gaussian variance σ^2 influence the performance most. The neural networks should have enough depth and width to approximate the underlying interactions, which can be checked by the performance on the validation dataset. The Gaussian variance σ^2 should incorporate the conditional distribution of the ground-truth state increment (learning target) into the posterior appropriately. As discussed in Sec. 4.6, we use grid search to determine these two hyperparameters. We empirically find a neural network with one hidden layer is enough to approximate the spring force, while it needs three hidden layers to approximate the electrical charge force and the forces in the crystallization simulation (Sec. 2.3). Detailed configurations for different experiments are summarized in Supplementary Table 2.

Supplementary Table 2: The neural network architecture and the Gaussian variance of CRI, Var-CRI and Evolving-CRI in different experiments.

Simulation	MLP layers in PIG'N'PI	Gaussian Variance σ^2
Spring	$[10, 256]$<br>$[256, 256]$<br>$[256, 2]$	0.1
Charge	$[10, 256]$<br>$[256, 256]$<br>$[256, 256]$<br>$[256, 256]$<br>$[256, 2]$	0.05
Crystallization	$[8, 300]$<br>$[300, 300]$<br>$[300, 300]$<br>$[300, 300]$<br>$[300, 2]$	0.001

3.2 Performance on the original simulation of NRI reference

As the particle simulation considered in this paper (See Sec. 2.2 and Sec. 4.5) is different from the original simulation provided by the NRI reference [8], we also tested CRI on the original simulations. The simulation of NRI can be generated by the associated code of NRI: `https://github.com/ethanfetaya/NRI/tree/master/data` or downloaded from `https://lis.csail.mit.edu/alet/neurips2019_data/`. To make a fair comparison, the generative module of CRI is exactly the same as NRI's decoder. We compute the interaction type distribution of every edge by marginalizing the joint distribution of all edges in each subgraph, allowing us to predict future states after several time steps in the same way as NRI. Further, for the experiment with charge data, we find this dataset contains extreme values caused by the pairwise force at small distances (r^{-2} in this case). We replace the Gaussian distribution which is the default setting of CRI (see Sec. 4.2) with the Laplacian distribution, avoiding the model focusing on these extreme values. CRI is trained to predict the state after 10 timesteps for the spring data with the variance hyperparameter equal to 0.0001 and predict the state at the next time step for the charge data with the variance hyperparameter equal to 0.001. Results are reported in Supplementary Table 3. The accuracy of the charge data clearly shows the superiority of CRI.

Supplementary Table 3: Accuracy (%) of edge type inference on the dataset provided by Kipf et al. [8]. The simulation contains five particles governed by the spring or charge force moving in a box. The performance values of NRI, MPM and ModularMeta are copied from their original reference. ModularMeta does not provide the standard derivation. The mean and standard derivation of CRI are computed from three independent experiments.

Model	Springs	Charged
NRI (Kipf et al. [8])	99.9 ± 0.0	82.1 ± 0.6
MPM (Chen et al. [9])	99.9 ± 0.0	93.3 ± 0.5
ModularMeta (Alet et al. [10])	99.9	88.4
CRI	99.9 ± 0.0	98.5 ± 0.4

3.3 Performance on causality discovery

Supplementary Table 4 contains the raw data used to report the results in Sec. 2.1.

Supplementary Table 4: Detailed results on causality discovery. The mean and standard deviation are computed from five experiments with different random initializations. (*) Indicates dataset for which we select the best performance of NRI among five random experiments, because some random seeds lead to severe sub-optimal performance.

Dataset	Method	Accuracy
VAR-a	NRI	0.5 (*)
	CRI	0.8985 ± 0.0137
VAR-b	NRI	0.9976 ± 0.0023
	CRI	0.9940 ± 0.0119
VAR-c	NRI	0.6670 (*)
	CRI	0.9996 ± 0.0001
Netsim	NRI	0.5512 ± 0.0457
	CRI	0.8021 ± 0.0451

3.4 Performance of Spring N5K2

Supplementary Table 5: Detailed results of different methods on Spring N5K2 in Sec. 2.2. The mean and the standard deviation are computed from five experiments.

	# training simulations	NRI original	NRI PIG'N'PI	MPM original	MPM PIG'N'PI	CRI	VarCRI
Relation accuracy	100	0.5015 ±0.0007	0.5889 ±0.0646	0.5144 ±0.0059	0.6246 ±0.0677	0.6935 ±0.0781	0.7294 ±0.0039
	500	0.5048 ±0.0051	0.6228 ±0.1066	0.5898 ±0.0745	0.7395 ±0.0026	0.9920 ±0.0004	0.9006 ±0.0024
	1000	0.5136 ±0.0086	0.7175 ±0.0794	0.8007 ±0.0437	0.7621 ±0.0016	0.9978 ±0.0003	0.9087 ±0.0013
	5000	0.8647 ±0.0763	0.8389 ±0.1014	0.9071 ±0.0037	0.7826 ±0.0027	0.9992 ±0.0003	0.8763 ±0.0792
	10000	0.9380 ±0.0016	0.9221 ±0.0052	0.9384 ±0.0008	0.8331 ±0.0095	0.9994 ±0.0007	0.8551 ±0.1106
MAE_{ef}	100	N/A	1.1696 ±0.2243	N/A	0.9753 ±0.1927	1.0011 ±0.2821	0.7726 ±0.0142
	500	N/A	1.0595 ±0.2836	N/A	0.7422 ±0.0018	0.1071 ±0.0024	0.1848 ±0.0042
	1000	N/A	0.8045 ±0.1629	N/A	0.7383 ±0.0023	0.0704 ±0.0055	0.1391 ±0.0084
	5000	N/A	0.2643 ±0.2368	N/A	0.7419 ±0.0005	0.0496 ±0.0064	0.1920 ±0.1906
	10000	N/A	0.0696 ±0.0048	N/A	0.5328 ±0.0443	0.0422 ±0.0113	0.2345 ±0.2609
MAE_{symm}	100	N/A	1.2744 ±0.5171	N/A	0.8422 ±0.5320	0.9835 ±0.8004	0.3342 ±0.0527
	500	N/A	0.8706 ±0.6217	N/A	0.0902 ±0.0063	0.0949 ±0.0105	0.2100 ±0.0121
	1000	N/A	0.3159 ±0.4459	N/A	0.0857 ±0.0042	0.0806 ±0.0020	0.1603 ±0.0079
	5000	N/A	0.3175 ±0.4182	N/A	0.0593 ±0.0023	0.0603 ±0.0054	0.2843 ±0.3336
	10000	N/A	0.0717 ±0.0069	N/A	0.1175 ±0.0086	0.0538 ±0.0129	0.3367 ±0.4001
After 1 step, MAE_{state}	100	0.0435 ±0.0010	0.0243 ±0.0016	0.0521 ±0.0040	0.0243 ±0.0020	0.0174 ±0.0016	0.0168 ±0.0002
	500	0.0321 ±0.0004	0.0213 ±0.0021	0.0344 ±0.0016	0.0190 ±3.4E-5	0.0029 ±0.0001	0.0063 ±0.0001
	1000	0.0275 ±0.0007	0.0191 ±0.0017	0.0253 ±0.0029	0.0183 ±4.3E-5	0.0019 ±0.0002	0.0052 ±0.0002
	5000	0.0081 ±0.0027	0.0078 ±0.0029	0.0105 ±0.0003	0.0177 ±2.3E-5	0.0013 ±0.0001	0.0045 ±0.0007
	10000	0.0034 ±0.0001	0.0038 ±0.0002	0.0064 ±0.0002	0.0141 ±0.0010	0.0012 ±0.0003	0.0047 ±0.0010
After 10 steps, MAE_{state}	100	0.4275 ±0.0095	0.2462 ±0.0154	0.5196 ±0.0424	0.2460 ±0.0210	0.1756 ±0.0153	0.1701 ±0.0020
	500	0.3143 ±0.0045	0.2139 ±0.0204	0.3379 ±0.0149	0.1927 ±0.0007	0.0285 ±0.0008	0.0625 ±0.0011
	1000	0.2689 ±0.0081	0.1930 ±0.0159	0.2504 ±0.0296	0.1854 ±0.0009	0.0181 ±0.0016	0.0520 ±0.0017
	5000	0.0786 ±0.0268	0.0769 ±0.0294	0.1024 ±0.0029	0.1791 ±0.0003	0.0124 ±0.0016	0.0453 ±0.0067
	10000	0.0327 ±0.0011	0.0369 ±0.0022	0.0616 ±0.0012	0.1426 ±0.0100	0.0109 ±0.0031	0.0466 ±0.0104

3.5 Performance of Spring N10K2

Supplementary Table 6: Detailed results on Spring N10K2 in Sec. 2.2. The mean and the standard deviation are computed from five experiments.

	# training simulations	NRI original	NRI PIG'N'PI	MPM original	MPM PIG'N'PI	CRI	VarCRI
Relation accuracy	100	0.5013 ±0.0006	0.5894 ±0.0548	0.5106 ±0.0044	0.6049 ±0.0271	0.5865 ±0.0481	0.5770 ±0.0537
	500	0.5019 ±0.0007	0.6283 ±0.0604	0.7079 ±0.0011	0.6385 ±0.0665	0.8692 ±0.0530	0.7357 ±0.0280
	1000	0.5011 ±0.0005	0.6419 ±0.0434	0.7255 ±0.0069	0.6756 ±0.0010	0.9607 ±0.0149	0.7655 ±0.0211
	5000	0.7557 ±0.0066	0.7482 ±0.0253	0.8012 ±0.0028	0.7042 ±0.0015	0.9745 ±0.0251	0.7587 ±0.0287
	10000	0.7996 ±0.0035	0.8018 ±0.0129	0.8283 ±0.0033	0.7848 ±0.0108	0.9857 ±0.0176	0.7845 ±0.0310
MAE_{ef}	100	N/A	1.0540 ±0.2994	N/A	0.9143 ±0.1596	1.3156 ±0.4488	1.2580 ±0.4071
	500	N/A	0.9083 ±0.2821	N/A	0.9150 ±0.2825	0.2211 ±0.0501	0.3312 ±0.0717
	1000	N/A	0.8330 ±0.1445	N/A	0.7747 ±0.0008	0.1288 ±0.0158	0.2545 ±0.0434
	5000	N/A	0.3493 ±0.0850	N/A	0.7504 ±0.0046	0.0976 ±0.0279	0.2497 ±0.0717
	10000	N/A	0.2306 ±0.0253	N/A	0.4254 ±0.0326	0.0706 ±0.0190	0.1727 ±0.0494
MAE_{symm}	100	N/A	0.5781 ±0.4118	N/A	0.4636 ±0.1866	1.0371 ±0.5573	0.9156 ±0.6001
	500	N/A	0.2245 ±0.2154	N/A	0.1152 ±0.0529	0.1808 ±0.0530	0.3846 ±0.1360
	1000	N/A	0.2845 ±0.3908	N/A	0.0603 ±0.0050	0.1029 ±0.0135	0.3156 ±0.0673
	5000	N/A	0.1666 ±0.0254	N/A	0.0594 ±0.0066	0.1218 ±0.0392	0.2819 ±0.0428
	10000	N/A	0.1206 ±0.0074	N/A	0.0870 ±0.0118	0.0885 ±0.0255	0.2379 ±0.1024
After 1 step, MAE_{state}	100	0.0794 ±0.0061	0.0348 ±0.0032	0.0609 ±0.0038	0.0371 ±0.0024	0.0230 ±0.0015	0.0248 ±0.0021
	500	0.0524 ±0.0018	0.0310 ±0.0023	0.0375 ±0.0009	0.0322 ±0.0025	0.0080 ±0.0012	0.0115 ±0.0008
	1000	0.0433 ±0.0013	0.0299 ±0.0012	0.0335 ±0.0014	0.0307 ±0.0000	0.0052 ±0.0006	0.0095 ±0.0006
	5000	0.0177 ±0.0005	0.0177 ±0.0019	0.0202 ±0.0006	0.0292 ±0.0001	0.0039 ±0.0008	0.0091 ±0.0007
	10000	0.0138 ±0.0004	0.0139 ±0.0007	0.0173 ±0.0003	0.0197 ±0.0008	0.0029 ±0.0006	0.0080 ±0.0006
After 10 steps, MAE_{state}	100	0.7616 ±0.0472	0.3506 ±0.0313	0.6032 ±0.0380	0.3773 ±0.0240	0.2292 ±0.0144	0.2476 ±0.0208
	500	0.5104 ±0.0164	0.3108 ±0.0230	0.3669 ±0.0105	0.3239 ±0.0250	0.0761 ±0.0119	0.1111 ±0.0076
	1000	0.4213 ±0.0123	0.2988 ±0.0119	0.3276 ±0.0142	0.3090 ±0.0003	0.0487 ±0.0060	0.0916 ±0.0064
	5000	0.1703 ±0.0041	0.1734 ±0.0194	0.1953 ±0.0057	0.2933 ±0.0015	0.0354 ±0.0082	0.0879 ±0.0073
	10000	0.1336 ±0.0037	0.1358 ±0.0069	0.1677 ±0.0037	0.1951 ±0.0077	0.0263 ±0.0060	0.0773 ±0.0059

3.6 Performance of the generalization

Supplementary Table 7: Detailed results on the generalization experiment in Sec. 2.2. The model is trained and selected on the training and validation dataset of Spring N5K2, and tested on Spring N10K2. The mean and the standard deviation are computed from five experiments.

	# training simulations	NRI original	NRI PIG'N'PI	MPM original	MPM PIG'N'PI	CRI	VarCRI
Relation accuracy	100	0.5015 ±0.0009	0.5472 ±0.0372	0.5050 ±0.0038	0.5667 ±0.0356	0.6118 ±0.0404	0.6404 ±0.0029
	500	0.5018 ±0.0012	0.5705 ±0.0638	0.5490 ±0.0338	0.6462 ±0.0027	0.9728 ±0.0027	0.7862 ±0.0036
	1000	0.5038 ±0.0029	0.6309 ±0.0454	0.6215 ±0.0632	0.6486 ±0.0029	0.9930 ±0.0017	0.8017 ±0.0026
	5000	0.6799 ±0.0337	0.6618 ±0.0461	0.6666 ±0.0558	0.6646 ±0.0017	0.9979 ±0.0008	0.7830 ±0.0643
	10000	0.7090 ±0.0022	0.6939 ±0.0037	0.6622 ±0.0640	0.6778 ±0.0034	0.9981 ±0.0022	0.7666 ±0.0883
$\mathrm{MAE}_{\mathrm{ef}}$	100	N/A	1.2026 ±0.2096	N/A	0.9420 ±0.0783	1.0352 ±0.2641	0.8126 ±0.0086
	500	N/A	1.0883 ±0.2601	N/A	0.8196 ±0.0011	0.1108 ±0.0024	0.1944 ±0.0050
	1000	N/A	0.8517 ±0.1497	N/A	0.7991 ±0.0016	0.0730 ±0.0056	0.1477 ±0.0107
	5000	N/A	0.2611 ±0.2108	N/A	0.7923 ±0.0002	0.0520 ±0.0067	0.1886 ±0.1719
	10000	N/A	0.0728 ±0.0052	N/A	0.7036 ±0.0118	0.0440 ±0.0116	0.2237 ±0.2345
$\mathrm{MAE}_{\mathrm{symm}}$	100	N/A	1.2917 ±0.5209	N/A	0.4164 ±0.2257	0.9810 ±0.7700	0.3507 ±0.0501
	500	N/A	0.8586 ±0.6092	N/A	0.0472 ±0.0040	0.0928 ±0.0092	0.2095 ±0.0138
	1000	N/A	0.3143 ±0.4333	N/A	0.0464 ±0.0036	0.0788 ±0.0019	0.1622 ±0.0124
	5000	N/A	0.2795 ±0.3569	N/A	0.0269 ±0.0020	0.0599 ±0.0045	0.2677 ±0.2990
	10000	N/A	0.0692 ±0.0065	N/A	0.0568 ±0.0037	0.0537 ±0.0122	0.3107 ±0.3545
After 1 step, $\mathrm{MAE}_{\mathrm{state}}$	100	1.0756 ±0.1297	0.0505 ±0.0013	0.1750 ±0.1299	0.0426 ±0.0022	0.0255 ±0.0013	0.0254 ±0.0003
	500	0.9439 ±0.1683	0.0477 ±0.0010	0.0850 ±0.0323	0.0374 ±0.0002	0.0051 ±0.0001	0.0101 ±0.0001
	1000	0.8576 ±0.1639	0.0486 ±0.0008	0.1684 ±0.1880	0.0361 ±0.0003	0.0033 ±0.0002	0.0089 ±0.0004
	5000	0.1096 ±0.0230	0.0397 ±0.0008	0.1172 ±0.1330	0.0350 ±0.0001	0.0024 ±0.0002	0.0081 ±0.0006
	10000	0.0553 ±0.0062	0.0402 ±0.0005	0.0653 ±0.0317	0.0330 ±0.0009	0.0021 ±0.0006	0.0080 ±0.0009
After 10 steps, $\mathrm{MAE}_{\mathrm{state}}$	100	10.3872 ±1.8296	0.5152 ±0.0165	1.8670 ±1.4760	0.4228 ±0.0199	0.2564 ±0.0118	0.2561 ±0.0039
	500	9.3614 ±1.8901	0.4864 ±0.0144	0.8496 ±0.3445	0.3682 ±0.0017	0.0473 ±0.0014	0.0978 ±0.0014
	1000	8.5264 ±1.8495	0.4990 ±0.0107	1.6630 ±1.8531	0.3581 ±0.0027	0.0302 ±0.0024	0.0861 ±0.0036
	5000	1.0734 ±0.2343	0.3911 ±0.0068	1.1360 ±1.2777	0.3472 ±0.0006	0.0208 ±0.0024	0.0780 ±0.0062
	10000	0.5400 ±0.0618	0.3958 ±0.0048	0.6397 ±0.3116	0.3258 ±0.0081	0.0187 ±0.0054	0.0774 ±0.0089

3.7 Performance of Spring N5K4

Supplementary Table 8: Detailed results on Spring N5K4 in Sec. 2.2. The mean and the standard deviation are computed from five experiments.

	# training simulations	NRI original	NRI PIG'N'PI	MPM original	MPM PIG'N'PI	CRI	VarCRI
Relation accuracy	100	0.2557 ±0.0014	0.2892 ±0.0115	0.2655 ±0.0043	0.2889 ±0.0085	0.2934 ±0.0089	0.2957 ±0.0156
	500	0.2565 ±0.0045	0.3182 ±0.0187	0.2788 ±0.0112	0.3287 ±0.0118	0.4907 ±0.0897	0.4319 ±0.0502
	1000	0.2617 ±0.0050	0.3103 ±0.0252	0.2859 ±0.0025	0.3348 ±0.0100	0.7097 ±0.1254	0.5238 ±0.0452
	5000	0.3921 ±0.0339	0.4134 ±0.0429	0.5217 ±0.0074	0.4736 ±0.0397	0.9467 ±0.0053	0.5778 ±0.0179
	10000	0.6159 ±0.0286	0.6245 ±0.0370	0.6256 ±0.0072	0.5521 ±0.0538	0.9428 ±0.0337	0.5873 ±0.0189
MAE_{ef}	100	N/A	1.8113 ±0.0986	N/A	1.7721 ±0.1245	2.9153 ±0.1560	2.3285 ±0.1863
	500	N/A	1.8265 ±0.1477	N/A	1.6031 ±0.0764	1.0668 ±0.2174	0.9792 ±0.1692
	1000	N/A	1.7421 ±0.1559	N/A	1.5552 ±0.0559	0.4657 ±0.3214	0.7141 ±0.2182
	5000	N/A	0.9647 ±0.1379	N/A	1.0271 ±0.1740	0.1018 ±0.0034	0.4897 ±0.0903
	10000	N/A	0.3121 ±0.2398	N/A	0.7910 ±0.1363	0.1054 ±0.0356	0.4872 ±0.0876
MAE_{symm}	100	N/A	2.1639 ±0.2470	N/A	1.5689 ±0.2592	4.4569 ±0.3673	3.3491 ±0.2548
	500	N/A	2.2274 ±0.3087	N/A	1.5879 ±0.2051	1.4356 ±0.3318	1.3564 ±0.1898
	1000	N/A	1.9968 ±0.4790	N/A	1.5371 ±0.0835	0.4719 ±0.3708	0.9243 ±0.3734
	5000	N/A	0.9231 ±0.2568	N/A	0.3014 ±0.0960	0.1339 ±0.0049	0.6212 ±0.1804
	10000	N/A	0.1602 ±0.0437	N/A	0.1810 ±0.0221	0.1439 ±0.0644	0.5434 ±0.0897
After 1 step, MAE_{state}	100	0.0569 ±0.0017	0.0317 ±0.0010	0.0548 ±0.0048	0.0350 ±0.0006	0.0210 ±0.0005	0.0219 ±0.0007
	500	0.0402 ±0.0009	0.0280 ±0.0006	0.0436 ±0.0024	0.0293 ±0.0003	0.0106 ±0.0016	0.0123 ±0.0005
	1000	0.0345 ±0.0004	0.0267 ±0.0005	0.0396 ±0.0016	0.0281 ±0.0002	0.0060 ±0.0019	0.0099 ±0.0010
	5000	0.0186 ±0.0011	0.0168 ±0.0021	0.0198 ±0.0005	0.0178 ±0.0012	0.0026 ±0.0001	0.0078 ±0.0004
	10000	0.0105 ±0.0003	0.0099 ±0.0003	0.0145 ±0.0004	0.0119 ±0.0014	0.0025 ±0.0005	0.0075 ±0.0001
After 10 steps, MAE_{state}	100	0.5604 ±0.0147	0.3188 ±0.0097	0.5456 ±0.0479	0.3540 ±0.0055	0.2090 ±0.0048	0.2199 ±0.0069
	500	0.3960 ±0.0082	0.2829 ±0.0062	0.4294 ±0.0219	0.2967 ±0.0033	0.1051 ±0.0157	0.1221 ±0.0052
	1000	0.3400 ±0.0046	0.2686 ±0.0044	0.3891 ±0.0156	0.2845 ±0.0026	0.0588 ±0.0190	0.0986 ±0.0106
	5000	0.1830 ±0.0105	0.1690 ±0.0213	0.1937 ±0.0052	0.1798 ±0.0126	0.0244 ±0.0008	0.0775 ±0.0042
	10000	0.1031 ±0.0030	0.0982 ±0.0028	0.1415 ±0.0038	0.1189 ±0.0145	0.0234 ±0.0049	0.0742 ±0.0012

3.8 Performance of Charge N5K2

Supplementary Table 9: Detailed results on Charge N5K2 in Sec. 2.2. NRI and MPM use the CNN reducer in their encoder, which is the default setting for the charge data in the original paper. The mean and the standard deviation are computed from five experiments.

	# training simulations	NRI original	NRI PIG'N'PI	MPM original	MPM PIG'N'PI	CRI	VarCRI
Relation accuracy	100	0.5037 ±0.0023	0.5046 ±0.0001	0.5043 ±0.0011	0.5065 ±0.0071	0.5041 ±0.0021	0.5027 ±0.0014
	500	0.5053 ±0.0033	0.5068 ±0.0031	0.5020 ±0.0015	0.5048 ±0.0018	0.9751 ±0.0060	0.9114 ±0.0111
	1000	0.5036 ±0.0008	0.5066 ±0.0040	0.5038 ±0.0026	0.5036 ±0.0026	0.9863 ±0.0009	0.9304 ±0.0045
	5000	0.5588 ±0.0508	0.6019 ±0.0238	0.5547 ±0.0433	0.5907 ±0.0326	0.9892 ±0.0006	0.9417 ±0.0011
	10000	0.6265 ±0.0180	0.6245 ±0.0058	0.5679 ±0.0652	0.6340 ±0.0219	0.9900 ±0.0009	0.9231 ±0.0409
MAE_{ef}	100	N/A	1.0972 ±0.0100	N/A	1.4087 ±0.0905	1.4059 ±0.0821	1.3416 ±0.0949
	500	N/A	1.1360 ±0.0199	N/A	1.5647 ±0.0217	0.0633 ±0.0103	0.0973 ±0.0177
	1000	N/A	1.1255 ±0.0198	N/A	1.6891 ±0.0474	0.0410 ±0.0034	0.0606 ±0.0044
	5000	N/A	0.6420 ±0.4259	N/A	0.4648 ±0.1760	0.0273 ±0.0011	0.0317 ±0.0043
	10000	N/A	0.3372 ±0.0385	N/A	0.2572 ±0.0462	0.0254 ±0.0005	0.0633 ±0.0680
MAE_{symm}	100	N/A	0.1990 ±0.0716	N/A	0.9261 ±0.3406	1.1143 ±0.1205	0.9894 ±0.2658
	500	N/A	0.4150 ±0.0857	N/A	1.3948 ±0.1108	0.0734 ±0.0122	0.1338 ±0.0230
	1000	N/A	0.3544 ±0.0981	N/A	1.8634 ±0.1109	0.0468 ±0.0032	0.0841 ±0.0069
	5000	N/A	0.8254 ±0.7952	N/A	0.2267 ±0.0353	0.0322 ±0.0013	0.0411 ±0.0032
	10000	N/A	0.2729 ±0.0533	N/A	0.1514 ±0.0305	0.0285 ±0.0015	0.0952 ±0.1180
After 1 step, MAE_{state}	100	0.0596 ±0.0021	0.0363 ±0.0003	0.0662 ±0.0041	0.0398 ±0.0002	0.0345 ±0.0001	0.0348 ±0.0001
	500	0.0517 ±0.0016	0.0372 ±0.0007	0.0569 ±0.0019	0.0397 ±0.0003	0.0018 ±0.0003	0.0031 ±0.0004
	1000	0.0480 ±0.0009	0.0375 ±0.0011	0.0558 ±0.0025	0.0391 ±0.0001	0.0012 ±0.0001	0.0022 ±0.0001
	5000	0.0254 ±0.0047	0.0152 ±0.0021	0.0539 ±0.0041	0.0167 ±0.0024	0.0006 ±0.0000	0.0011 ±0.0001
	10000	0.0134 ±0.0009	0.0118 ±0.0010	0.0398 ±0.0087	0.0100 ±0.0017	0.0006 ±0.0000	0.0019 ±0.0018
After 10 steps, MAE_{state}	100	0.5405 ±0.0177	0.3339 ±0.0031	0.6072 ±0.0459	0.3619 ±0.0028	0.3133 ±0.0015	0.3160 ±0.0015
	500	0.4657 ±0.0139	0.3426 ±0.0077	0.5207 ±0.0237	0.3531 ±0.0018	0.0163 ±0.0028	0.0289 ±0.0038
	1000	0.4296 ±0.0087	0.3456 ±0.0114	0.5094 ±0.0240	0.3464 ±0.0013	0.0106 ±0.0009	0.0202 ±0.0008
	5000	0.2261 ±0.0390	0.1470 ±0.0177	0.4935 ±0.0369	0.1596 ±0.0194	0.0063 ±0.0002	0.0107 ±0.0008
	10000	0.1241 ±0.0090	0.1155 ±0.0085	0.3663 ±0.0785	0.1006 ±0.0172	0.0057 ±0.0000	0.0182 ±0.0171

3.9 Performance of the crystallization experiment with the evolving graph topology

Supplementary Table 10: Interpolation performance on learning heterogeneous interactions in the crystallization simulation in Sec. 2.3. The mean and the standard deviation are computed from five experiments.

Metrics	NRI original	NRI PIG'N'PI	MPM original	MPM PIG'N'PI	Evolving-CRI
Relation accuracy	0.5173 ±0.0128	0.8047 ±0.0759	0.5156 ±0.0063	0.5254 ±0.0096	0.9420 ±0.0071
MAE_{ef}	N/A	3.7966 ±1.2645	N/A	14.7288 ±0.1398	0.2259 ±0.0156
MAE_{symm}	N/A	5.0660 ±1.9263	N/A	10.4671 ±0.4191	0.2953 ±0.0237
$MAE_{acceleration}$	36.5034 ±1.2649	9.5760 ±2.0994	48.1343 ±1.6359	44.0858 ±0.0570	0.5806 ±0.0513

Supplementary Table 11: Extrapolation performance on learning heterogeneous interactions in the crystallization simulation in Sec. 2.3. The mean and the standard deviation are computed from five experiments.

Metrics	NRI original	NRI PIG'N'PI	MPM original	MPM PIG'N'PI	Evolving-CRI
Relation accuracy	0.5859 ±0.0464	0.6241 ±0.0767	0.5488 ±0.0636	0.5502 ±0.0350	0.8658 ±0.0404
MAE_{ef}	N/A	11.4696 ±4.4449	N/A	14.1664 ±0.2484	0.8568 ±0.2342
MAE_{symm}	N/A	9.7949 ±7.6433	N/A	5.8915 ±0.9859	0.8319 ±0.2828
$MAE_{acceleration}$	47.1006 ±0.2720	30.6527 ±14.4418	47.2572 ±0.3354	47.5451 ±0.1873	4.0430 ±1.0426

3.10 Ablation study of CRI

We are interested in knowing whether the inference module or the generative module of CRI contributes most to its performance. Supplementary Figure 2 reports the prediction accuracy on Spring N5K2. The results show that **CRI+GNN** is close to **CRI+PIG'N'PI**. This demonstrates that only changing the generative module does not have any considerable effect on the prediction performance. However, the performance of **NRI+PIG'N'PI** has a significant gap with **CRI+PIG'N'PI**. This shows that the proposed *collective* relational inference method rather than the generative module is the key element for the relational inference. It further suggests that the proposed CRI framework could be applied to other tasks (*e.g.*, the causality discovery) where the standard message-passing GNN or other kinds of GNN should be used as the generative module.

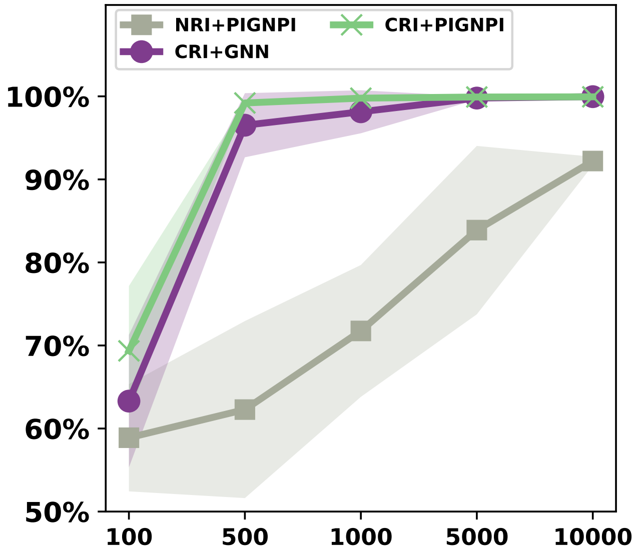

Supplementary Figure 2: The prediction accuracy on Spring N5K2. **CRI+PIGNPI** stands for the proposed CRI framework with the PIG'N'PI as the generative module. **CRI+GNN** stands for the proposed CRI framework with the standard graph neural network as the generative module. **NRI+PIGNPI** stands for the NRI framework with PIG'N'PI as the generative module. The mean and standard derivation are computed from five independent experiments.

3.11 Performance on a noisy dataset

We add noise to the Spring N5K2 dataset with 500 training examples, 1000 validation and 1000 testing examples, to evaluate the robustness of the proposed CRI. We impose white noise on the observed particle positions at each time step to simulate measurement noise. Then, we compute velocities and accelerations from the noisy position. We adopt the following equation to impose noise on the measured positions:

$$\tilde{r}_{i,d}^{t} \leftarrow r_{i,d}^{t} + \beta X_{i,d}^{t}$$

where $\tilde{r}_{i,d}^{t}$ is the d-th dimension of the noisy position of particle i at time t, $X_{i,d}^{t} \sim \mathcal{N}(0,1)$ is the random number sampled independently from the standard normal distribution and β is a constant controlling the magnitude of the noise.

Different values for β will change both inputs (position and velocity) and the learning target (acceleration in this case). Here, we define the noise level as the **average relative change of the target (state increment)**:

$$\text{noise level} = \frac{1}{T}\frac{1}{|V|}\frac{1}{d}\sum_{t=1}^{T}\sum_{i\in V}\sum_{k\in d}\frac{|\tilde{a}_{i,k}^{t} - a_{i,k}^{t}|}{|a_{i,k}^{t}|} \tag{14}$$

where $\tilde{a}_{i,k}^{t}$ is the k-th dimension of the noisy acceleration of particle i at time t. We test 1e-7, 1e-6, 1e-5, 1e-4 and 1e-3 as the values for β. The performances of CRI with different noise levels are summarized in Supplementary Table 12. We find that CRI is robust to a certain level of noise. Interestingly, the performance becomes slightly better with small noise (*e.g.*, $\beta =$1e-7). However, it fails to infer the heterogeneous interactions with severe noise (*e.g.*, $\beta =$1e-3).

Supplementary Table 12: Noise level for different β.

β	noise level	Accuracy	MAE_{ef}	MAE_{symm}	$\text{MAE}_{\text{state}}$ 1 step	$\text{MAE}_{\text{state}}$ 10 step
0.0	0.0	0.9920 ±0.0004	0.1071 ± 0.0024	0.0949 ± 0.0105	0.0029 ± 0.0001	0.0285 ± 0.0008
1e-7	0.0250	0.9930 ±0.0017	0.1017 ± 0.0064	0.0998 ± 0.0126	0.0028 ± 0.0002	0.0274 ± 0.0019
1e-6	0.3816	0.9924 ±0.0021	0.1051 ± 0.0069	0.0934 ± 0.0027	0.0030 ± 0.0002	0.0284 ± 0.0023
1e-5	4.3715	0.9929 ±0.0016	0.1009 ± 0.0074	0.0967 ± 0.0092	0.0051 ± 0.0001	0.0272 ± 0.0019
1e-4	36.9642	0.9900 ±0.0021	0.1125 ± 0.0105	0.1003 ± 0.0119	0.0393 ± 0.0000	0.0477 ± 0.0015
1e-3	277.8361	0.5128 ±0.0159	23.1797 ± 3.4117	40.7052 ± 9.9086	0.3799 ± 0.0012	0.6010 ± 0.0355

3.12 Experiment with evolving graph topology in terms of the cutoff radius

In the main text, we assume that we have access to the ground-truth cutoff interaction radius in cases where particles do not interact with all other particles. Here, we investigate how this value influences the performance of Evolving-CRI. We take the same experiment in Sec. 2.3 but vary the number of incoming interactions (which is equivalent to varying the cutoff radius) of each particle to construct the computational graph for Evolving-CRI. We train the generative module of Evolving-CRI with different numbers of incoming interactions. After training, we apply the trained model to predict the interaction type of those edges appearing in the testing data of the simulation. We report the accuracy of interpolation prediction in Supplementary Fig. 3 and Supplementary Table 13. Our results show that the accuracy increases as we increase the cutoff radius (number_of_neighbors = 2, 3, 4, 5) until we reach the ground-truth cutoff radius (number_of_neighbors = 5)

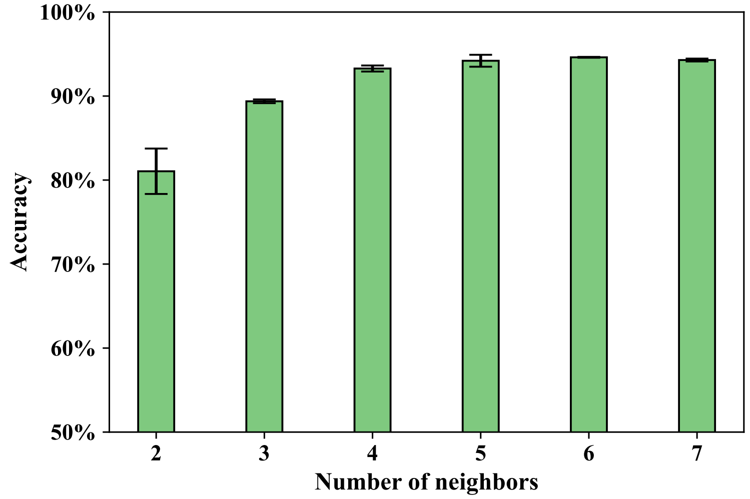

Supplementary Figure 3: Accuracy of edge type inference in terms of the number of incoming interactions. In data generation, each particle is influenced by the nearest five particles. We use the same dataset splits in Sec. 2.3 to train, valid and test Evolving-CRI, and report the interpolation prediction accuracy. The mean and standard derivation of Evolving-CRI are computed from three independent experiments.

Supplementary Table 13: Accuracy (%) of edge type inference in terms of the number of incoming interactions. This table contains the values in Supplementary Fig. 3.

#Incoming interactions	Accuracy
2	81.0 ± 2.7
3	89.4 ± 0.2
4	93.3 ± 0.4
5 $\leftarrow$ underlying simulation	94.2 ± 0.7
6	94.6 ± 0.1
7	94.3 ± 0.2

3.13 Force prediction visualization

We show the visualization of the predicted spring and charge force fields in Supplementary Fig. 4 and Supplementary Fig. 5. These visualizations further demonstrate the strength of CRI whose predictions are closer to the ground-truth.

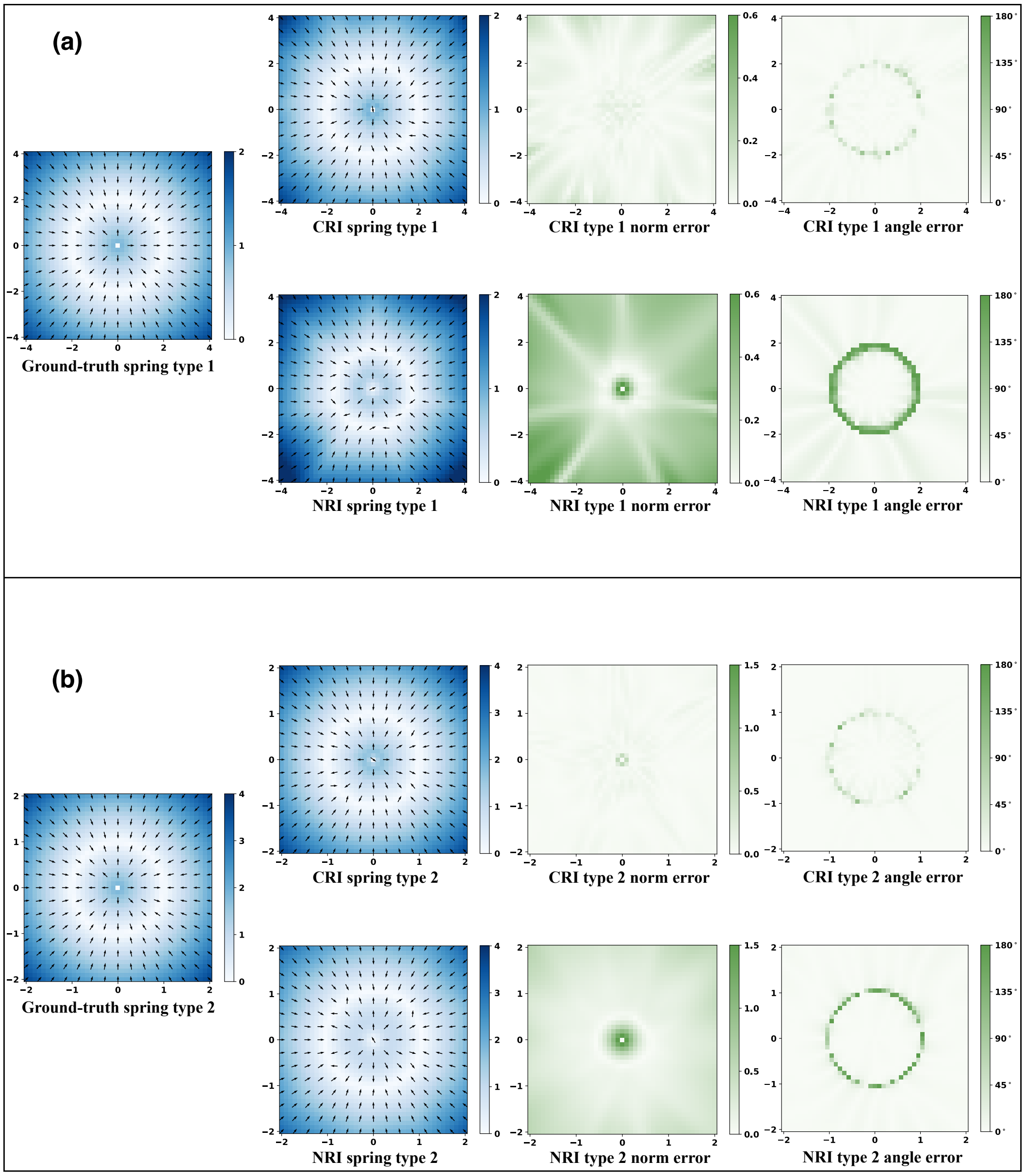

Supplementary Figure 4: Comparison between the ground-truth **spring** force field and the predicted spring force fields by CRI and NRI. We visualize these two different types of forces in the blue figures of (a) and (b) by fixing a particle in the center, moving another particle and plotting the force of the moving particle at different positions. The color indicates force magnitude. The vector indicates the force direction. The green figures are the magnitude and angle errors of the predicted force and the ground-truth force. The predicted force of NRI and CRI are computed by the corresponding best models trained on Spring N10K2 with 10k training examples.

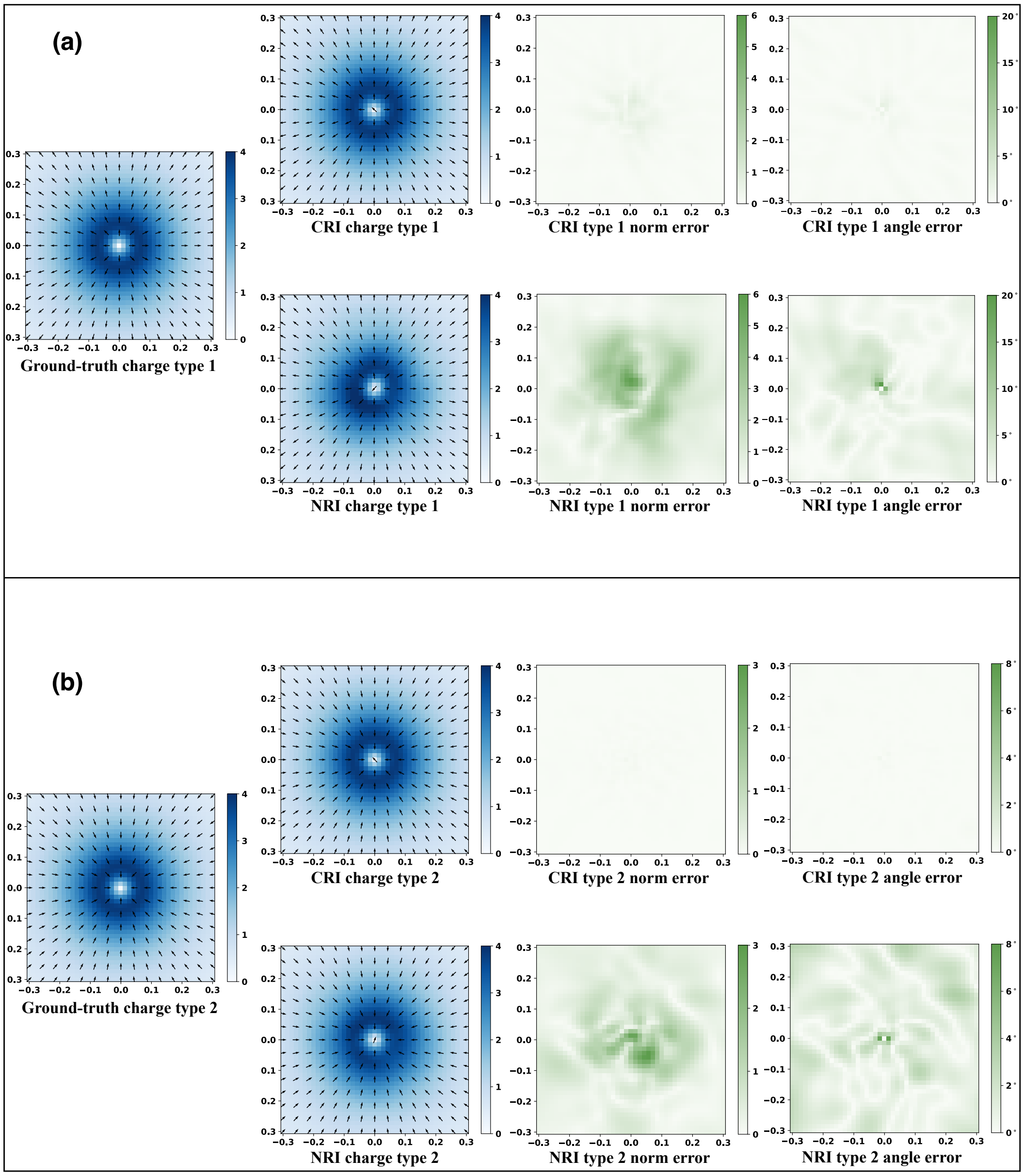

Supplementary Figure 5: Comparison between the ground-truth **charge** force field and the predicted spring force fields by CRI and NRI. We visualize these two different types of forces in the blue figures of (a) and (b) by fixing a particle in the center, moving another particle and plotting the force of the moving particle at different positions. The color indicates force magnitude. The vector indicates the force direction. The green figures are the magnitude and angle errors of the predicted force and the ground-truth force. The predicted force of NRI and CRI are computed by the corresponding best models trained on Charge N5K2 with 10k training examples.

3.14 Symbolic regression

In addition to predicting the values of pairwise interactions (*e.g.*, forces) with the trained model, it can also be used as input to an arbitrary symbolic regression method to find the underlying equation. Here, we apply the off-the-shelf Deep Symbolic Optimization (DSO) [1], which is one of the start-of-the-art symbolic regression methods, on the prediction made by the trained models for the spring and charge systems. The discovered equations are summarized in Supplementary Table 14. The results show that the discovered equations based on the trained CRI are very close to the ground-truth equations. Further, we want to emphasize that the outcome of CRI is not limited to a specific symbolic regression algorithm but offers the potential for any symbolic regression method to more effectively discover the equation based on the developed CRI method.

Supplementary Table 14: Equation discovery by applying Deep Symbolic Optimization (DSO). Ground-truth stands for the underlying equation used in the simulations when generating the dataset. SR+GT stands for directly using the ground-truth pairwise force as the input target for DSO. SR+CRI corresponds to using the predicted pairwise force by CRI as the input target for DSO. SR+NRI corresponds to using the predicted pairwise force by NRI as the input target for DSO.

Dataset	Method	Equation
Spring K2	Ground-truth	$0.5 * (r - 2)$ $2 * (r - 1)$
	SR + GT	$0.500 * r - 1.000$ $2.000 * r - 2.000$
	SR + CRI	$0.503 * r - 1.007$ $2.005 * r - 2.006$
	SR + NRI	$0.408 * r - 0.673$ $r * (r - 1.718)$
Charge K2	Ground-truth	$\frac{r}{(r^2 + 0.01)^{3/2}}$ $-\frac{r}{(r^2 + 0.01)^{3/2}}$
	SR + GT	$\frac{0.948}{r * (r - 0.036) + 0.020}$ $-\frac{0.948}{r * (r - 0.036) + 0.020}$
	SR + CRI	$\frac{0.937}{r * (r - 0.040) + 0.020}$ $-\frac{0.943}{r * (r - 0.037) + 0.020}$
	SR + NRI	$-\frac{0.542}{r * (0.131 - r) - 0.029}$ $-\frac{0.838}{r * (r - 0.065) + 0.020}$

[1] `https://github.com/brendenpetersen/deep-symbolic-optimization`

Supplementary References

[1] Dimitris G. Tzikas, Aristidis C. Likas, and Nikolaos P. Galatsanos. The variational approximation for bayesian inference. *IEEE Signal Processing Magazine*, 25(6):131–146, 2008. doi: 10.1109/MSP.2008.929620.

[2] Greg C. G. Wei and Martin A. Tanner. A monte carlo implementation of the em algorithm and the poor man's data augmentation algorithms. *Journal of the American statistical Association*, 85(411):699–704, 1990. doi: 10.1080/01621459.1990.10474930.

[3] Christopher M. Bishop. *Pattern Recognition and Machine Learning (Information Science and Statistics)*. Springer-Verlag, Berlin, Heidelberg, 2006. ISBN 0387310738.

[4] Rajesh Ranganath, Dustin Tran, and David M. Blei. Hierarchical variational models. In Maria Florina Balcan and Kilian Q. Weinberger, editors, *Proceedings of the 33rd International Conference on International Conference on Machine Learning (ICML)*, volume 48, pages 324–333. JMLR, 2016.

[5] Samy Bengio and Yoshua Bengio. Taking on the curse of dimensionality in joint distributions using neural networks. *IEEE Transactions on Neural Networks*, 11(3):550–557, 2000.

[6] Christian P. Robert and George Casella. *Monte Carlo Statistical Methods (Springer Texts in Statistics)*, volume 2. Springer-Verlag, Berlin, Heidelberg, 2005. ISBN 0387212396.

[7] C.F. Jeff Wu. On the convergence properties of the EM algorithm. *The Annals of statistics*, pages 95–103, 1983.

[8] Thomas Kipf, Ethan Fetaya, Kuan-Chieh Wang, Max Welling, and Richard Zemel. Neural relational inference for interacting systems. In Jennifer Dy and Andreas Krause, editors, *Proceedings of the 35th International Conference on Machine Learning*, volume 80, pages 2688–2697, Stockholm, Sweden, 10–15 Jul 2018. PMLR.

[9] Siyuan Chen, Jiahai Wang, and Guoqing Li. Neural relational inference with efficient message passing mechanisms. In *Proceedings of the 35th AAAI Conference on Artificial Intelligence*, volume 35, pages 7055–7063. AAAI Press, 2021.

[10] Ferran Alet, Erica Weng, Tomás Lozano Pérez, and Leslie Pack Kaelbling. Neural relational inference with fast modular meta-learning. In H. Wallach, H. Larochelle, A. Beygelzimer, F. d'Alché-Buc, E. Fox, and R. Garnett, editors, *Proceedings of the 33rd International Conference on Neural Information Processing Systems (NeurIPS)*, volume 32, pages 11827–11838. Curran Associates, Inc., 2019.
